# A novel and effective ECG method to differentiate right from left ventricular outflow tract arrhythmias: Angle-corrected V2S

**DOI:** 10.3389/fcvm.2022.868634

**Published:** 2022-10-13

**Authors:** Shifeng Qiu, Zhuhua Sun, Xinzhong Li, Jianyong Li, Xiaobo Huang, Menghui Liu, Jianping Bin, Yulin Liao, Jiancheng Xiu, Daogang Zha, Yumei Xue, Lichun Wang, Yuegang Wang

**Affiliations:** ^1^Department of Cardiology, Nanfang Hospital, Southern Medical University, Guangzhou, China; ^2^Guangdong Provincial Key Laboratory of Shock and Microcirculation, Nanfang Hospital, Southern Medical University, Guangzhou, China; ^3^State Key Laboratory of Organ Failure Research, Nanfang Hospital, Southern Medical University, Guangzhou, China; ^4^Department of Health Management, The Third Affiliated Hospital of Southern Medical University, Guangzhou, China; ^5^Department of Cardiology, The First Affiliated Hospital of Sun Yat-sen University, Guangzhou, China; ^6^Key Laboratory on Assisted Circulation, Ministry of Health, Guangzhou, China; ^7^Guangdong Cardiovascular Institute, Guangdong Provincial People’s Hospital, Guangdong Academy of Medical Sciences, Guangzhou, China; ^8^Guangdong Provincial Key Laboratory of Clinical Pharmacology, Guangdong Provincial People’s Hospital, Guangdong Academy of Medical Sciences, Guangzhou, China

**Keywords:** radiofrequency ablation, electrocardiogram, V2S, angle, ventricular outflow tract arrhythmias, cardiac long-axis

## Abstract

**Background and aims:**

Standard 12-lead electrocardiogram (ECG) patterns combined with the anatomical cardiac long-axis angle revealed by chest X-ray can prevent the influence of cardiac rotation, physical shape, and lead position, so it may be an ideal means to predict the origin of the outflow tract (OT) ventricular arrhythmias (OTVAs) for ablation procedures. The study explores the value of this strategy in identifying the origin of OTVA.

**Methods:**

This study was conducted using a retrospective cohort and a prospective cohort of consecutive patients at two centers. The anatomical cardiac long-axis angle was calculated by measuring the angle between the cardiac long-axis (a line joining the apex to the midpoint of the mitral annulus) and the horizontal plane on a chest X-ray. The V2S angle was calculated as the V2S amplitude times the angle. We ultimately enrolled 147 patients with symptomatic OTVAs who underwent successful radiofrequency catheter ablation (RFCA) (98 women (66.7%); mean age 46.9 ± 14.7 years; 126 right ventricular OT (RVOT) origins, 21 left ventricular OT (LVOT) origins) as a development cohort. The new algorithm was validated in 48 prospective patients (12 men (25.0%); mean age 48.0 ± 15.8 years; 36 RVOT, 12 LVOT origins).

**Results:**

Patients with RVOT VAs had greater V2S, long-axis angle, and V2S angle than patients with LVOT VA (all *P* < 0.001). The cut-off V2S angle obtained by receiver operating characteristic (ROC) curve analysis was 58.28 mV° for the prediction of RVOT origin (sensitivity: 85.7%; specificity: 95.2%; positive predictive value: 99.1%; negative predictive value: 52.6%). The AUC achieved using the V2S angle was 0.888 (*P* < 0.001), which was the highest among all indexes (V2S/V3R: 0.887 (*P* < 0.016); TZ index: 0.858 (*P* < 0.001); V1-2 SRd: 0.876 (*P* < 0.001); V3 transition: 0.651 (*P* < 0.001)). In the prospective cohort, the V2S angle had a high overall accuracy of 93.8% and decreased the procedure time (*P* = 0.002).

**Conclusion:**

V2S angle can be a novel measure that can be used to accurately differentiate RVOT from LVOT origins. It could help decrease ablation duration and radiation exposure.

## Introduction

Outflow tract (OT) ventricular arrhythmias (OTVAs), mainly composed of premature ventricular complexes (PVCs) or ventricular tachycardia (VT), are commonly encountered and sometimes harmful, as they can cause cardiomyopathy leading to dangerous conditions such as heart failure and sudden cardiac death (1, 2). Catheter ablation is a common curative therapy for OTVA patients with and without structural heart disease when drugs are ineffective or have unacceptable side effects (3, 4). Accurate prediction of a right ventricular outflow tract (RVOT) vs. left ventricular outflow tract (LVOT) origin of OTVA can direct the catheter ablation strategy, thereby reducing ablation duration and avoiding operative complications (5–7). Current algorithms that only rely on the standard 12-lead electrocardiogram (ECG) to identify OTVAs are limited to the cardiac rotation caused by the physical shape and cannot achieve the desired accuracy (8–10).

The amplitude and precordial transition of the 12-lead ECG depends on the cardiac depolarization vector axis and are influenced by the cardiac rotation and lead position. Even though the vectorcardiogram (VCG) can reflect electrical activation more accurately than scalar ECG in certain circumstances, this non-anatomical recording is still prone to error and takes more time (11, 12). Chest X-ray is simple and convenient in the clinic; more importantly, this imaging method can reflect anatomical characteristics and cardiac transposition and can be used to correct the recording differences caused by anatomical variations to a certain extent (13). Therefore, we hypothesized that 12-lead ECG combined with the anatomical cardiac long-axis calculated from the chest X-ray could more precisely predict the origin of OTVA.

In this study, we aimed first to develop a novel algorithm that took into account the anatomical cardiac long-axis plus 12-lead ECG to differentiate RVOT- from LVOT-origin VA. Second, we compared this algorithm with earlier methods of identifying OTVA and compared their accuracy in predicting LVOT vs. RVOT origin in a prospective cohort.

## Materials and methods

The data, analytic methods, and study materials can be made available to other researchers for purposes of reproducing the results or replicating the procedure. This trial was approved by the Human Research Ethics Committee of Nanfang Hospital of Southern Medical University and the First Affiliated Hospital of Sun Yat-sen University (NFEC-2020-083).

### Study design

This study was done in 2 parts: 1) a review and analysis of a retrospective cohort of premature ventricular contraction (PVC)/ventricular tachycardia (VT) patients who underwent successful radiofrequency catheter ablation (RFCA), whose aim was to develop a new diagnostic method to distinguish LVOT- from RVOT-origin PVC/VT, which we used to identify the origin of the interventricular septum and the coronary sinuses; and 2) an analysis of a prospective cohort for evaluating the validity of the new diagnostic method.

### Study population

Consecutive patients who were indicated for OVTA ablation with ECG left bundle branch block (LBBB) morphology and transition in leads V2-V4 without structural abnormalities were recruited in two centers. Atypical LBBB morphologies, such as “annular” or “ostial” morphologies, were excluded. Patients with coronary heart disease, structural heart disease, paced rhythm, arrhythmogenic right ventricular cardiomyopathy, failed ablation, left ventricular ejection fraction (LVEF) of < 50%, conduction disturbances in the underlying sinus rhythm, or a secondary cause of arrhythmias were excluded from the study. Additionally, those with a secondary possible cause of arrhythmias, such as electrolyte disturbance, impaired kidney, liver or thyroid function, or hematologic or rheumatological diseases, were excluded.

### Electrocardiographic assessment

A 2-min standard surface 12-lead ECG was recorded during sinus rhythm and during PVC/VT at 25 mm/s speed (10 mm/mv amplitude) with chest and limb leads placed in standard positions in all patients. The ECG recording system was the FX-7402 (Fukuda Denshi, Inc., Tokyo, Japan) or the LabSystem Pro (Boston Scientific, Inc.). The morphologies of the sinus beats and OTVAs were analyzed on the same 12-lead ECG using electronic calipers. The following parameters were measured from the surface ECG of the first beat of PVC/VT: the S-wave amplitude in leads V1-V3, R-wave amplitude in leads V1-V3, and total QRS duration. Then, all the parameters above were normalized to the anatomical cardiac long-axis. The apex is clearly defined anatomically; the exact point used to locate the base has varied between studies, but the most convenient has been the atrioventricular rings (14). To determine the long-axis of the heart, we took a line from the center of the mitral valvar orifice to the left ventricular apex (13). The anatomical cardiac long-axis can be estimated by measuring the angle between the cardiac long-axis anatomically and the horizontal plane on chest radiography. In general, the cardiac long-axis angle is different in people of different physiques, in which taller, thinner people have an angle greater than 45 degrees (°), while shorter, heavier people have an angle less than 45° (13).

**FIGURE 1:**
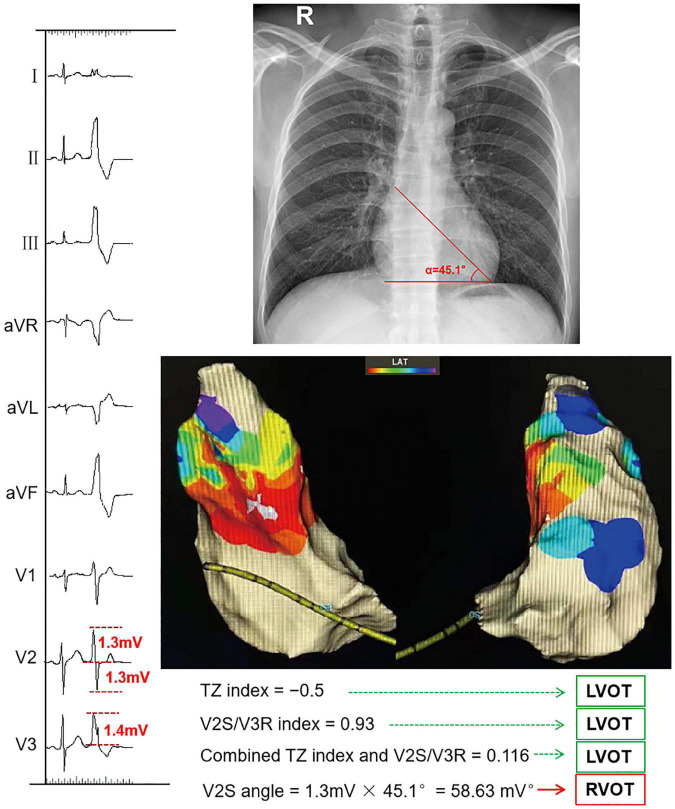
Example of calculating the V2S angle and several ECG algorithms. The TZ index was –0.5 according to the following formula: TZ score of OTVA minus TZ score of sinus beat (2–2.5 = –0.5). The V2S/V3R index was calculated with the following formula: V2S/V3R = 1.3 mV/1.4 mV = 0.93. The combined TZ index and V2S/V3R were calculated with the formula *Y* = –1.15 × (TZ)–0.494 × (V2S/V3R) = –1.15 × (–0.5)–0.494 × 0.93 = 0.116. These indexes indicate that this OTVA had a left ventricular outflow tract (LVOT) origin, though it was successfully ablated in the septal aspect of the right ventricular outflow tract (RVOT). The measurements of the V2S angle during the OTVAs are as follows: the S-wave amplitude in lead V2 is 1.3 mV. The angle (α) between the cardiac long-axis anatomically and the horizontal plane on chest radiography is 45.1°. The V2S angle was calculated with the following formula: (V2S × α) = 1.3 mv × 45.1° = 58.63 (mV°). The V2S angle was verified as accurate.

In our study, the S-wave in lead V2 of PVC/VT normalized to the cardiac long-axis, for calculating the angle practically, was termed the angle-corrected V2S (hereafter, V2S angle). Similarly, the S-wave in leads V1 and V3 and the R-wave in leads V1-V3 of PVC/VT normalized to the cardiac long-axis were termed the V1S angle, V3S angle, V1R angle, V2R angle, and V3R angle, respectively. For example, the V2S angle was calculated from the 12-lead surface ECG with this formula: V2S amplitude × angle (mV°) (Figure 1). QRS duration was measured in the lead with the widest QRS complex on the 12-lead surface ECG (15).

We compared our novel ECG criterion with several published ECG algorithms: 1) TZ index (16) = TZ score of the OTVA minus the TZ score of the sinus beat. The TZ score was graded in 0.5-point increments according to the site of the R-wave transition (e.g., TZ in V2 = 2 points, V2–V3 = 2.5 points, V3 = 3 points, and V3–V4 = 3.5 points). The cut-off value of the TZ index obtained by receiver operating characteristic (ROC) curve analysis was ≥ 0 for the prediction of RVOT origin. 2) V2S/V3R (10): the S-wave amplitude in lead V2 divided by the R-wave amplitude in lead V3 during the OTVA. The cut-off value of the V2S/V3R index obtained by ROC curve analysis was > 1.5 mV for the prediction of RVOT origin. 3) The combined TZ index and V2S/V3R (8) were calculated with this formula: *Y* = –1.15 × (TZ)–0.494 × (V2S/V3R). If the TZ index and V2S/V3R are ≥ –0.76, it predicts LVOT origin; 4) V1–V2 S-R difference (V1-2 SRd) (mV) (17): The S-R difference in V1-V2 on the 12-lead surface ECG was calculated with this formula: (V1S amplitude + V2S amplitude) – (V1R amplitude + V2R amplitude). If V1–2 SRd > 1.625 mV, it predicts an RVOT origin. 5) V3 transition (18): An R-wave deflection interval in V3 (> 80 ms) and an R-wave amplitude index in V1 (> 0.3) predict an LVOT origin. 6) R wave amplitude in lead I (9): R amplitude ≥ 0.1 mV in lead I predicts an LVOT origin. 7) Initial r wave surface area (ISA) index (19): This index is measured by multiplying the R wave duration in milliseconds by the R wave amplitude in mV in the V1 (ISA V1) or V2 lead (ISA V1). If ISA ≥ 15, it predicts an LVOT origin. 8) V1R/V1S index (20): The R-wave amplitude is divided by the S-wave amplitude in the V1 lead during the OTVAs. An R-wave duration index ≥ 50% and an R/S-wave amplitude index ≥ 30% strongly suggest an ASC origin in patients with a typical LBBB morphology and an inferior axis.

Then, all ECG algorithms above were corrected for the anatomical cardiac long-axis. The TZ index corrected for the cardiac long-axis, for calculating by the angle practically, was termed the angle-corrected TZ index (hereafter, TZ index angle). The other corrected ones were termed V2S/V3R angle, combined TZ index and V2S/V3R angle, V1-2 SRd angle, V3 transition angle, R wave amplitude in lead I angle, ISA angle, and V1R/V1S angle.

All measurements were performed by 2 electrophysiologists who were blinded to the final diagnosis and the site of origin to eliminate interobserver variability and bias. Measurements were independently performed and reviewed by 3 physicians who were blinded to the PVC/VT site of origin.

### Ablation protocol

All antiarrhythmic medications were suspended at least five half-lives before the ablation procedure. The ablation procedure was performed without sedation. Conventional ablation was performed in 130 (88.4%) patients using a Marin 7 Fr 4-mm deflectable tip catheter (Medtronic Inc., Minneapolis, Minnesota, USA) with an Atak II RF generator (Medtronic Inc) and the Ensite NavX electroanatomic mapping system (St. Jude Medical Inc., St Paul, Minnesota, USA). The ablation was performed in temperature control mode with a target temperature of 43°C and a power limit of 40 W in the RVOT and LVOT (for 50 s) and with a target temperature of 43°C and a power limit of 40 W in the aortic root (for 30–45 s). VA ablation was performed with the CARTO electroanatomic mapping system (Biosense Webster, Diamond Bar, California, USA) in 17 (11.6%) patients to guide the ablation. In these patients, a 3.5-mm irrigated-tip catheter (Navistar, Biosense-Webster, Diamond Bar, California, USA) was used for mapping and ablation. Target ablation sites were chosen from the combination of activation mapping and pace mapping as described previously (21). If ablation failed, another target was chosen for ablation. Mapping and ablation of the LVOT area were performed *via* a retrograde aortic approach with a 100 IU/kg heparin bolus. If necessary, more doses were administered to maintain an activated clotting time of 250–350 s. Acute procedural ablation success was defined as no spontaneous or induced clinical PVCs occurring within 30 min after the last radiofrequency energy application.

### Statistical analyses

Statistical analyses were conducted using SPSS, version 19.0 (SPSS Inc., Chicago, IL, USA). Data are expressed as mean ± SD for continuous variables and as percentages for categorical variables. The Shapiro–Wilk test was used to test normality, in which a *P*-value > 0.05 indicated normally distributed data. Continuous variables that showed a normal distribution were compared using Student’s *t*-test and ANOVA, whereas the Mann–Whitney *U* test and Kruskal–Wallis test were used for normally distributed samples. Categorical variables are expressed as numbers (percentage) and were compared using the chi-square test, except for those with *n* ≤ 5 for 1 or more expected values, which were compared using Fisher’s exact test. Multiple linear regression analysis was used to calculate the partial regression coefficient β and the 95% confidence interval (CI) for ECG characteristics and the angle.

Statistical significance was defined as a *P*-value < 0.05 for all comparisons. Pearson’s and Spearman’s correlation coefficients were calculated to examine the relationships between continuous variables. ROC curve analysis was performed to determine the cut-off value of the new diagnostic metric for differentiating LVOT from RVOT origins and calculate the sensitivity, specificity, positive predictive value (PPV), and negative predictive value (NPV).

## Results

First, we retrospectively enrolled 178 patients with symptomatic frequent PVC/VT who underwent successful RFCA at Nanfang Hospital of Southern Medical University and The First Affiliated Hospital of Sun Yat-sen University hospitalized from February 2017 to May 2019, except for the patients who met the exclusion criteria below. We excluded 13 patients who originated from the tricuspid annulus or mitral annulus. Patients with underlying bundle branch block (*n* = 3), paced rhythm (*n* = 1), and failed ablation (*n* = 3) were excluded. Eleven patients who underwent redo procedures were also excluded. Therefore, 147 patients were successfully enrolled (49 men (33.3%); mean age 46.9 ± 14.7 years, 126 RVOT, 21 LVOT origins), all of whom underwent *de novo* ablation. ECG and exercise stress testing or coronary angiography demonstrated no evidence of structural heart disease in any patient, and all antiarrhythmic drugs were discontinued for at least 5 half-lives before the study. The local ethics committee approved the study protocol, and each patient gave written informed consent.

Second, an analysis of the prospective cohort for evaluating the validity of the new diagnostic method was performed in 48 patients from May 2019 to January 2020 (12 men (25.0%); mean age 48.0 ± 15.8 years, 36 RVOT, 12 LVOT origins).

The baseline characteristics of patients, including age, sex, body mass index (BMI), QRS duration, PVC burden of 24 h, documented episodes of VT, prior history of ablation, LVEF, and angle, were recorded for all patients (Table 1). LVEF was assessed using Simpson’s equation in the apical 4-chamber view. The procedure time referred to the time of the operation from the beginning to the end of the patient on the operating table.

**TABLE 1:** Baseline characteristics of patients in retrospective and prospective cohorts.

	Retrospective cohort				Prospective cohort			
	All	RVOT	LVOT	*P*-value	All	RVOT	LVOT	*P*-value
Patients, n (%)	147	126 (85.8)	21 (14.2)	NA	48	36 (75.0)	12 (25.0)	NA
Age (y)	46.9 ± 14.7	45.6 ± 14.8	54.8 ± 12.1	0.004	48.0 ± 15.8	47.8 ± 15.6	48.8 ± 17.1	0.860
Male, n (%)	49 (33.3)	43 (34.1)	6 (28.6)	0.803	12 (25.0)	6 (16.7)	6 (50.0)	0.021
BMI, n (kg/m^2^)	21.8 ± 2.3	22.0 ± 2.3	21.0 ± 2.3	0.072	20.8 ± 4.0	20.6 ± 4.3	21.5 ± 2.4	0.534
LVEF, n (%)	61.4 ± 9.1	61.9 ± 9.3	58.2 ± 7.3	0.080	63.1 ± 5.1	63.0 ± 5.2	63.6 ± 5.1	0.714
PVC burden, /24-h Holter (%)	21.5 ± 9.8	21.7 ± 10.4	20.3 ± 4.9	0.556	22.3 ± 10.5	21.0 ± 8.1	26.0 ± 15.6	0.154
Angle, n (°)	37.6 ± 8.5	38.6 ± 8.5	31.7 ± 5.9	<0.001	36.5 ± 8.8	38.8 ± 8.3	29.7 ± 6.4	0.001
**Clinical arrhythmia, n (%)**								
Frequent PVC	99 (67.3)	85 (67.5)	14 (66.7)	0.715	37 (77.1)	28 (77.8)	9 (75.0)	0.847
Non-SVT	13 (8.9)	11 (8.7)	2 (9.5)	0.346	3 (6.3)	2 (5.6)	1 (8.3)	0.737
Sustained VT	35 (23.8)	30 (23.8)	5 (23.8)	0.859	8 (16.7)	6 (16.7)	2 (16.7)	1.000
Prior RFCA	21 (14.3)	19 (15.1)	2 (9.5)	0.929	2 (4.2)	1 (2.8)	1 (8.3)	0.415

Data are expressed as mean ± SD. BMI, body mass index; LVEF, left ventricular ejection fraction; TZ, transitional zone; RVOT, right ventricular outflow tract; LVOT, left ventricular outflow tract; PVC, premature ventricular contraction; VT, ventricular tachycardia; V1-2 SRd, V1–V2 S-R difference.

### Retrospective cohort analysis and development of a new diagnostic method

The baseline characteristics of 147 patients (49 men (33.3%); mean age 46.9 ± 14.7 years, 126 RVOT, 21 LVOT origins) are compared in Table 2. All patients were successfully ablated. Among patients with LVOT VA, all of the successful ablation sites were localized in the left or right coronary sinus. Among patients with RVOT VA, all of the successful ablation sites were localized in the interventricular septum RVOT.

**TABLE 2:** Baseline patient characteristics and electrocardiographic characteristics of the retrospective cohort (*n* = 147).

	RVOT (*n* = 126)	LVOT (*n* = 21)	*P*-value	AUC	Cut-off	95% confidence interval		*P*-value
Age (years)	46 ± 15	55 ± 12	0.004					
Sex (Male, %)	43 (34.1)	6 (28.6)	0.803					
BMI (kg/m^2^)	22.0 ± 2.3	21.0 ± 2.3	0.072					
LVEF (%)	61.9 ± 9.3	58.2 ± 7.3	0.080					
QRS duration (ms)	157.6 ± 20.6	150.8 ± 13.6	0.057					
PVC burden (%/24-h Holter)	21.7 ± 10.4	20.3 ± 4.9	0.556					
Patients had documented episodes of VT (n)	0.32 ± 0.79	0.29 ± 0.56	0.859					
Prior ablation history (n)	0.15 ± 0.36	0.14 ± 0.48	0.929					
**ECG and angle related indicators**								
V1S (mv)	1.26 ± 0.49	0.69 ± 0.32	<0.001	0.821	0.78	0.755	0.888	<0.001
V2S (mv)	1.82 ± 0.79	1.04 ± 0.59	<0.001	0.776	1.28	0.675	0.877	<0.001
V3S (mv)	0.93 ± 0.75	0.52 ± 0.56	0.018	0.693	0.63	0.571	0.816	0.005
V1R (mv)	0.26 ± 0.20	0.36 ± 0.22	0.030	0.362	0.85	0.239	0.485	<0.001
V2R (mv)	0.40 ± 0.33	0.91 ± 0.53	<0.001	0.183	2.30	0.085	0.280	<0.001
V3R (mv)	0.62 ± 0.43	1.46 ± 0.74	<0.001	0.143	4.30	0.059	0.228	<0.001
angle (°)	38.6 ± 8.5	31.7 ± 5.9	<0.001	0.750	31.5	0.638	0.862	<0.001
V1S angle (mV°)	44.95 ± 25.34	21.65 ± 9.78	<0.001	0.824	31.77	0.757	0.890	<0.001
V2S angle (mV°)	68.64 ± 29.85	31.80 ± 17.08	<0.001	0.888	58.28	0.831	0.945	<0.001
V3S angle (mV°)	35.18 ± 27.91	15.87 ± 16.73	<0.001	0.736	18.14	0.624	0.847	0.001
V1R angle (mV°)	9.82 ± 8.42	11.87 ± 8.58	0.306	0.425	9.15	0.297	0.554	0.274
V2R angle (mV°)	14.76 ± 10.96	29.95 ± 21.06	<0.001	0.247	91.20	0.128	0.365	0.061
V3R angle (mV°)	22.89 ± 14.47	48.08 ± 30.05	<0.001	0.205	136.30	0.098	0.313	<0.001
**Several ECG algorithms**								
V2S/V3R	4.81 ± 6.74	1.08 ± 1.05	0.013	0.887	1.40	0.812	0.961	<0.001
TZ index	0.43 ± 0.89	−0.79 ± 0.96	<0.001	0.858	−0.25	0.757	0.958	<0.001
Combined TZ index and V2S/V3R	−2.87 ± 3.55	0.37 ± 1.45	<0.001	0.088	−13.68	0.007	0.169	<0.001
V1-2 SRd (mv)	2.42 ± 1.08	0.46 ± 1.29	<0.001	0.876	0.73	0.795	0.957	<0.001
V3 transition	0.97 ± 0.18	0.67 ± 0.48	<0.001	0.651	0.50	0.505	0.796	0.027
R wave amplitude in lead I	28.87 ± 23.46	31.42 ± 22.73	0.644	0.457	2.67	0.315	0.598	0.524
ISA	22.35 ± 34.66	57.49 ± 40.28	<0.001	0.186	250	0.091	0.281	<0.001
ISA V1	2.11 ± 2.52	0.78 ± 1.16	0.019	0.723	0.36	0.605	0.841	0.001
ISA V2	0.36 ± 2.02	3.53 ± 9.28	0.001	0.151	42	0.045	0.257	<0.001
V1R/V1S index	0.39 ± 1.40	3.85 ± 11.40	0.001	0.313	−1.00	0.193	0.433	0.006

Data are expressed as mean ± SD. BMI, body mass index; LVEF, left ventricular ejection fraction; TZ, transitional zone; RVOT, right ventricular outflow tract; LVOT, left ventricular outflow tract; PVC, premature ventricular contraction; VT, ventricular tachycardia; V1-2 SRd, V1–V2 S-R difference.

There were no significant differences in sex (*χ^2^* = 0.361, *P* = 0.803) or LVEF (*P* = 0.080) between the two groups. Patients with RVOT VA were significantly younger (*P* < 0.05) than patients with LVOT VA. The V2S was found to be significantly higher in RVOT origins than in LVOT origins (*P* < 0.001). The angle (*P* < 0.001) and the V2S angle (*P* < 0.001) were found to be significantly greater in RVOT origins than in LVOT origins. There was no significant difference in BMI, QRS duration, PVC burden at 24 h, documented episodes of VT, or prior history of ablation between the two groups (all *P* > 0.05) (Table 2).

### Linear regression analysis for ECG characteristics and angle

**FIGURE 2:**
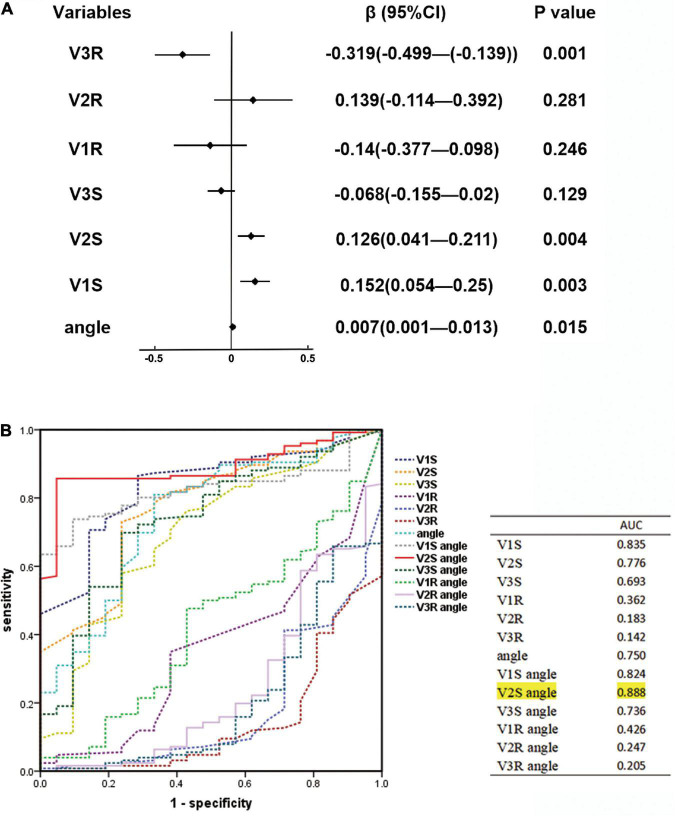
**(A)** Linear regression analysis for ECG characteristics and angle. The regression coefficient β and 95% CI of each independent variable are shown above. The final multiple linear regression model was statistically significant (*F* = 14.278, *P* < 0.001), and 38.9% of the variation in the dependent variable ECG algorithm could be explained by V1S, V2S, V3R, and angle (adjusted *R*^2^ = 0.389). **(B)** Receiver operating characteristic (ROC) curve analysis for anatomic prediction of RVOT origin was also performed. The area under the curve (AUC) of the V2S angle was 0.888, which was the highest among all indicators.

This study explored the effects of V1S, V2S, V3S, V1R, V2R, V3R, and angle on the ECG algorithm that we used to differentiate RVOT from LVOT origins by applying multiple linear regression analysis. The final multiple linear regression model constructed was statistically significant (*F* = 14.278, *P* < 0.001), and 38.9% of the variation in the dependent variable ECG algorithm could be explained by V1S, V2S, V3R, and angle (adjusted *R*^2^ = 0.389). The regression coefficient β and 95% CI of each independent variable are shown in Figure 2A. ROC curve analysis for anatomic prediction of an RVOT origin was also studied. The area under the curve (AUC) of the V2S angle was 0.888, which was the highest among all studied indicators (Figure 2B).

### Receiver operating characteristic curve analysis to determine the predictive value of the V2S angle for differentiating left ventricular outflow tract from right ventricular outflow tract

**FIGURE 3:**
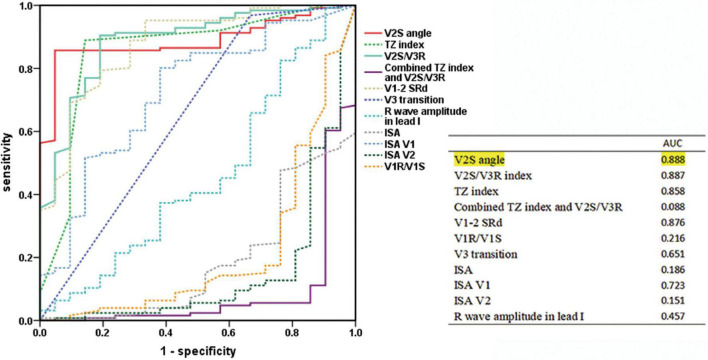
Predictive accuracy of the V2S angle and several ECG algorithms of previous studies. ROC curve analysis for anatomic prediction of RVOT origin. The AUC of the V2S angle was 0.888, which was the highest of all.

By analyzing the predictive accuracy of R- and S-wave amplitudes in leads V1 to V3 during PVC/VT multiplied by the angle in 147 patients, we found that the V2S angle was the best index. The AUC of the V2S angle was 0.888 (*P* < 0.001). In addition, the AUC of the V2S angle was greater than that of the TZ index (AUC = 0.887), V2S/V3R (AUC = 0.858), and V1-2 SRd (AUC = 0.876) (Figure 3).

**FIGURE 4:**
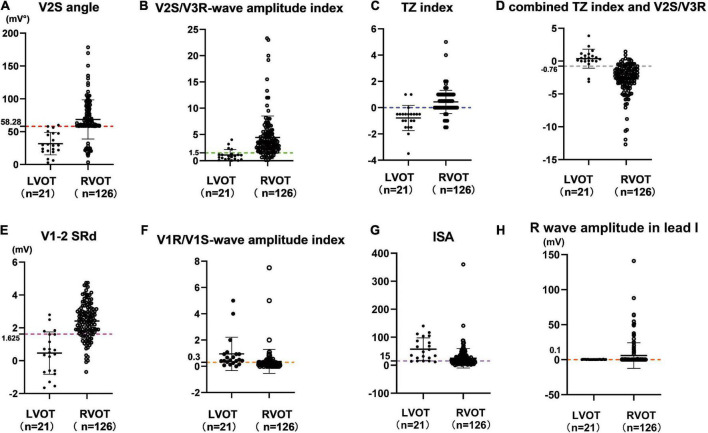
Scatterplot diagram of the V2S angle **(A)** and several ECG algorithms **(B–H)** between ventricular arrhythmias from RVOT and LVOT. The red dotted line indicates the optimal cut-off value of the V2S angle. The V2S angle of 58.28 mV° predicted the RVOT origin with 85.7% sensitivity and 95.2% specificity (PPV: 99.1%; NPV: 52.6%). Other diagnostic indexes for predicting RVOT origin (sensitivity, specificity, PPV, NPV) are shown in Table 3.

The cut-off value of the V2S angle obtained by ROC curve analysis was 58.28 mV° for the prediction of RVOT origin (sensitivity: 85.7%; specificity: 95.2%; PPV: 99.1%; NPV: 52.6%) (Table 3), and the 95% confidence interval of the AUC was 0.831–0.945 (Table 2). A scatterplot diagram of the V2S angle against RVOT and LVOT origins is shown in Figure 4A.

**TABLE 3:** Diagnostic indexes of ECG criteria for predicting RVOT origin.

	Sensitivity	Specificity	PPV	NPV
V2S angle ≥ 58.28 mV°	85.7%	95.2%	99.1%	52.6%
V2S/V3R > 1.5	89.7%	81.0%	96.6%	56.7%
TZ index ≥ 0	88.9%	81.0%	96.6%	54.8%
combined TZ index and V2S/V3R <−0.76	86.5%	23.8%	87.2%	22.7%
V1-2 SRd > 1.625	81.7%	28.6%	87.3%	20.7%
R wave amplitude in lead I ≥ 2.67 mV	99.2%	9.5%	52.3%	92.2%
ISA ≥ 250	0.8%	100.0%	100%	50.2%
ISA V1 ≥ 0.36	80.2%	61.9%	67.8%	75.8%
ISA V2 ≥ 42	0	100.0%	0	50.0%
V1R/V1S ≤ 0.3	82.5%	23.8%	86.7%	18.5%

PPV, positive predictive value; NPV, negative predictive value; BMI, body mass index; LVEF, left ventricular ejection fraction; TZ, transitional zone; RVOT, right ventricular outflow tract; LVOT, left ventricular outflow tract; PVC, premature ventricular contraction; VT, ventricular tachycardia; V1-2 SRd, V1–V2 S-R difference.

### Retrospective cohort analysis of several angle-corrected ECG algorithms

**FIGURE 5:**
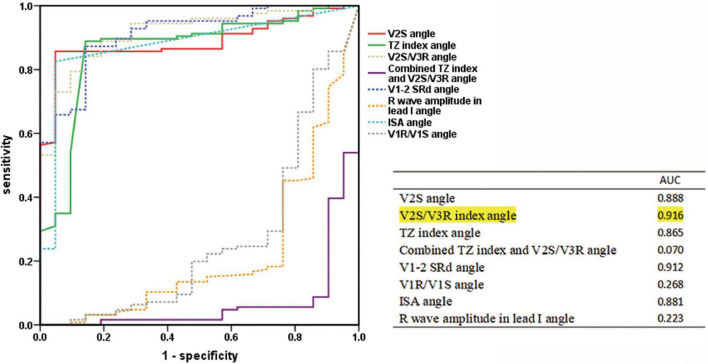
Receiver operating characteristic curve analysis of the V2S angle and the angle-corrected ECG algorithms. The predictive accuracy of several ECG algorithms was improved by correcting with the angle. The AUC of the V2S/V3R index angle (AUC = 0.916) was the highest of all, even greater than that of the V2S angle (AUC = 0.888).

In the retrospective cohort (*n* = 147), several ECG algorithms from previous studies were corrected for the angle. Surprisingly, the AUCs of the angle-corrected ECG algorithms for differentiating LVOT from RVOT were all higher than those before correction (V2S/V3R angle vs. V2S/V3R = 0.916 vs. 0.887; the TZ index angle vs. the TZ index = 0.865 vs. 0.858; V1-2 SRd angle vs. V1-2 SRd = 0.912 vs. 0.876, etc.) (Table 4). The AUC of the V2S/V3R angle (AUC = 0.916) was the best, even greater than that of the V2S angle (AUC = 0.888) (Figure 5).

**TABLE 4:** The angle-corrected ECG algorithms applied to the retrospective cohort (*n* = 147).

	RVOT (*n* = 126)	LVOT (*n* = 21)	*P*-value	AUC	Cut-off	95% confidence interval		*P*-value
V2S/V3R angle (°)	186.18 ± 255.09	32.36 ± 2.73	<0.001	0.916	48.36	0.860	0.971	<0.001
TZ index angle (°)	17.30 ± 32.51	−27.93 ± 38.35	<0.001	0.865	−5.68	0.778	0.953	<0.001
Combined TZ index and V2S/V3R angle (°)	−111.87 ± 135.81	16.13 ± 52.10	<0.001	0.070	−508.28	0.009	0.132	<0.001
V1-2 SRd angle (mv°)	92.65 ± 43.86	11.62 ± 41.47	<0.001	0.912	52.71	0.853	0.971	<0.001
R wave amplitude in lead I angle (mv°)	0.13 ± 0.17	0.36 ± 0.30	<0.001	0.223	0.98	0.106	0.340	<0.001
ISA angle	9.33 ± 8.34	0.64 ± 2.92	<0.001	0.882	1.31	0.805	0.956	<0.001
V1R/V1S angle (°)	13.90 ± 46.48	119.80 ± 353.95	0.001	0.475	1565.00	0.338	0.612	0.717

Data are expressed as mean ± SD. BMI, body mass index; LVEF, left ventricular ejection fraction; TZ, transitional zone; RVOT, right ventricular outflow tract; LVOT, left ventricular outflow tract; PVC, premature ventricular contraction; VT, ventricular tachycardia; V1-2 SRd, V1–V2 S-R difference.

### Prospective cohort analysis and validation of the new diagnostic method

A total of 48 consecutive patients with PVC/VT (12 men (25.0%); mean age 48.0 ± 15.8 years, 36 RVOT, 12 LVOT origins) with LBBB who underwent successful RFCA at Nanfang Hospital of Southern Medical University constituted the prospective cohort to test the validity of the new diagnostic method.

As in the retrospective cohort, there were no significant differences in baseline clinical characteristics between the 2 groups of PVC/VT origin (*P* > 0.05) in the prospective cohort, except there were more men in the LVOT group (*n* = 6, 50.0%) than in the RVOT group (*n* = 6, 16.7%) (*χ^2^* = 5.333, *P* = 0.021). The detailed clinical characteristics of the prospective cohort are described in Table 5. Compared with LVOT origins, the V2S was found to be significantly higher (*P* = 0.019), the angle was significantly greater (*P* = 0.001), and the V2S angle was significantly greater in RVOT origins (*P* = 0.001).

**TABLE 5:** Patient characteristics and electrocardiographic characteristics in the prospective cohort (*n* = 48).

	RVOT (*n* = 36)	LVOT (*n* = 12)	*P*-value
Age (years)	48 ± 16	49 ± 17	0.860
Sex (Male, %)	6 (16.7)	6 (50.0)	0.021
BMI (kg/m^2^)	20.6 ± 4.3	21.5 ± 2.4	0.534
LVEF (%)	63.0 ± 5.2	63.6 ± 5.1	0.714
V2S (mv)	2.21 ± 0.76	1.53 ± 1.06	0.019
QRS duration (ms)	153.1 ± 24.5	150.8 ± 24.3	0.786
PVC burden before RF (%/24-h Holter)	21.0 ± 8.1	26.0 ± 15.6	0.154
PVC burden after RF (%/24-h Holter)	0.2 ± 1.1	0.4 ± 1.2	0.649
Patient had any documented episode of VT (n)	0.25 ± 0.65	0.25 ± 0.62	1.000
Prior ablation history (n)	0.03 ± 0.17	0.08 ± 0.29	0.415
angle (°)	38.8 ± 8.3	29.7 ± 6.4	0.001
V2S angle (mV°)	84.49 ± 36.38	43.26 ± 33.55	0.001
V2S/V3R	3.46 ± 3.71	1.20 ± 1.69	0.048
TZ index	0.53 ± 0.99	−0.63 ± 0.53	<0.001

Data are expressed as mean ± SD. BMI, body mass index; LVEF, left ventricular ejection fraction; TZ, transitional zone; RVOT, right ventricular outflow tract; LVOT, left ventricular outflow tract; PVC, premature ventricular contraction; VT, ventricular tachycardia; V1-2 SRd, V1–V2 S-R difference.

**FIGURE 6:**
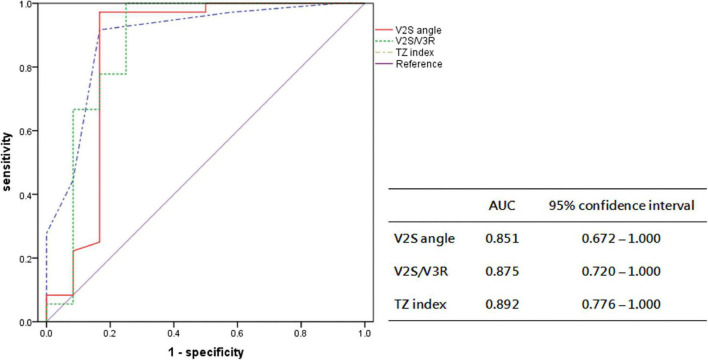
Receiver operating characteristic curve analysis for the anatomic prediction of OTVA origin with V2S angle and previous indexes in the prospective cohort. The AUC of V2S angle was 0.851, similar to that of V2S/V3R (0.875) and the TZ index (0.892).

In the prospective cohort, the sensitivity, specificity, PPV, and NPV of the V2S angle for predicting the RVOT origin over the LVOT origin were 97.2%, 83.3%, 94.6%, and 90.9%, respectively. The new diagnostic method had a high overall accuracy of 93.8% (Figure 6).

### The differences in duration of the ablation procedure in the retrospective vs. prospective cohort

**FIGURE 7:**
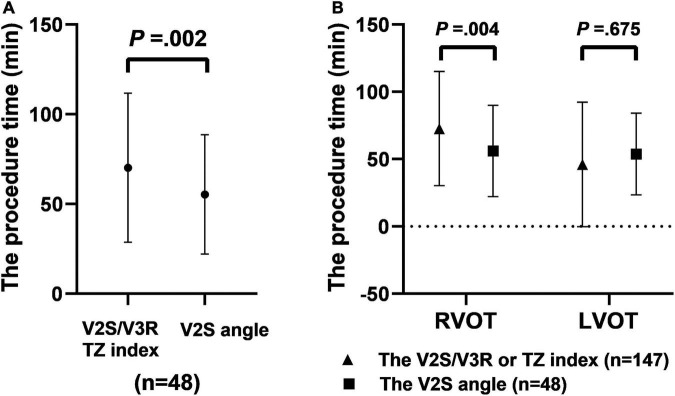
The procedure time when using different diagnostic indexes made up of ECG criteria for predicting RVOT origin. The procedure time with the V2S angle was shorter than with V2S/V3R or the TZ index before RFCA in the prospective cohort (*n* = 48) (*P* = 0.002) **(A)**. The procedure time in the prospective cohort (*n* = 48) with the V2S angle was shorter than that in the retrospective cohort (*n* = 147) with V2S/V3R or the TZ index **(B)**.

Overall, the procedure time in the prospective cohort (*n* = 48) (78.8 ± 31.8 min) was significantly shorter than that in the retrospective cohort (*n* = 147) (99.5 ± 40.8 min) due to the analysis of the V2S angle before RFCA (*P* = 0.002) (Figure 7A).

Specifically, for predicting RVOT origin, the procedure time in the prospective cohort in which the V2S angle was used (*n* = 36) (80.0 ± 32.0 min) was significantly shorter than that in the retrospective cohort in which the V2S/V3R or the TZ index was used before RFCA (*n* = 128) (102.6 ± 42.6 min) (*P* = 0.004). For predicting LVOT origin, the procedure time in the prospective cohort in which the V2S angle was used (*n* = 12) (75.2 ± 32.3 min) was shorter than that in the retrospective cohort in which the V2S/V3R or the TZ index was used (*n* = 19) (78.7 ± 13.3 min), but the difference was not significant (*P* = 0.675) (Figure 7B).

## Discussion

### Main findings

In the current study, we developed a novel ECG criterion, the angle-corrected V2S, with an AUC of 0.88, making it the first ECG criterion proven to be predictive of LVOT and RVOT origins of VA. However, the V2S angle is only used to identify the origin of the interventricular septum and the coronary sinuses. The main finding of the present study is that a V2S angle ≥ 58.28 mV° predicted an RVOT origin with a sensitivity of 85.7% and specificity of 95.2% in the overall analysis (PPV: 99.1%, NPV: 52.6%). This means that the specific cut-off for differentiating LVOT from RVOT arrhythmias was confirmed in the prospective cohort. Compared with previous methods, such as 12-lead ECG and VCG, it is more accurate and time-saving and may be an ideal strategy for identifying OTVA. In addition, as reported previously, the V2S was found to be significantly higher in RVOT origins than LVOT origins in the present study (*P* < 0.001). The anatomical cardiac long-axis was found to be significantly greater in RVOT origins than LVOT origins (*P* < 0.001), which means that VA originating from the RVOT may be more likely to occur in taller, thinner people with a smaller angle on chest radiography.

### Anatomical considerations and the V2S angle

The information provided by any particular form of electrocardiography obviously depends on the electrode configuration (number and positions of the electrodes utilized to sample the body surface potential distribution in space and time) (11). Anatomically, the aortic root occupies a central location within the heart, and the RVOT is located anteriorly and left of the aortic root (22). In patients whose hearts are normally positioned, the RVOT is located anterior to the LVOT at the level of the aortic cusps. The pulmonary valves are positioned approximately 1–2 cm superior to the aortic valves (23). Nikoo et al. (19) showed that the ISA index, defined as the highest value calculated in the V1 or V2 lead by multiplying the R wave duration in milliseconds by the R wave amplitude in terms of mV, is highly accurate and the most specific compared with other previously reported indexes in differentiating left from right outflow tract-originated VT/PVC. Regarding this anatomical background, V1 and V2 are the closest chest leads to the LVOT and RVOT, which are the best derivations to distinguish OTVAs. The closer a focus of an impulse is to a lead, the greater the degree of S-wave amplitude in that lead. (17) This contiguity can be thought to result in higher R-wave amplitude and lower S-wave amplitude in the frontal leads V1 and V2 during OTVAs originating from the LVOT than those originating from the RVOT (24, 25). Therefore, it would be reasonable to develop an ECG algorithm using the S-wave amplitude in lead V1 or V2.

However, the location of the heart within the thorax varies significantly between individuals, which means that leads V1 and V2 are affected by different physiques. Analyzing previous research, such as on V2S/V3R and the TZ index, we speculate that the V2S amplitude is more meaningful than the V1S amplitude, taking into account the long cardiac axis. Compared with lead V1, lead V2 is the least influenced by the physique. Therefore, the V2S would be the best chest lead to differentiate LVOT and RVOT in both taller, thinner and shorter, heavier people. Additionally, chest radiography can reflect anatomical characteristics and cardiac transposition and can be used to correct the recording differences caused by anatomical variations to a certain extent (13). Thus, through ECG and chest X-ray, the anatomical position of the heart can be located better and more accurately, and the deviation can be reduced as much as possible. The anatomical position of the heart can be reflected by locating the anatomical cardiac long-axis. To some extent, the use of the anatomical cardiac long-axis can correct for some factors, like the patient’s physique, cardiac rotation, and respiratory variation. The anatomical cardiac long-axis can be reflected by measuring the angle between the cardiac long-axis anatomically and the horizontal plane on chest radiography. In brief, the long-axis angle can partly reflect the anatomical rotation and the position of the heart with its projection in the three spatial planes, thus giving rise to the vectorcardiographic loops in the respective planes (26). For example, the heart is overhanging in taller, thinner patients, so the angle on their chest X-ray is greater. If the heart is rotated clockwise, both the angle and the V2S amplitude will decrease. Accordingly, the V2S angle would be the best index among all indexes anatomically.

### Comparison with the previous indexes

Outflow tract ventricular arrhythmias mostly originate from the RVOT (27). Many studies have aimed to localize OTVAs. A combined model using the TZ index and V2S/V3R would be more accurate, but its clinical utility may be limited (28). We compared our criteria to previous indexes in our population. The cut-off value of the V2S/V3R index obtained by ROC curve analysis was 1.40 for the prediction of RVOT origin (sensitivity: 89.7%, specificity: 81.0%, PPV: 96.6%, NPV: 56.7%), and its AUC was 0.887 (*P* < 0.001). The cut-off value of the TZ index obtained by ROC curve analysis was −0.25 for the prediction of RVOT origin (sensitivity: 88.9%, specificity: 81.0%, PPV: 96.6%, NPV: 54.8%), and its AUC was 0.858 (*P* < 0.001). There was no significant bias in the patient population of this study compared with those reported previously. The AUC of the V1-2 SRd was 0.876 (*P* < 0.001). The AUC of the V3 transition was 0.651 (*P* < 0.001). The combined TZ index and V2S/V3R (AUC = 0.088), the IAS (AUC = 0.186), and the V1R/V1S index (AUC = 0.313) were used for the prediction of LVOT origin. The cut-off value of the V2S angle obtained by ROC curve analysis was 58.28 mV° for the prediction of RVOT origin (sensitivity: 85.7%, specificity: 95.2%, PPV: 99.1%, NPV: 52.6%), and its AUC was 0.888 (*P* < 0.001) (Tables 2, 3). Scatterplot diagrams of several ECG algorithms between ventricular arrhythmias from RVOT and LVOT are shown in Figures 4B–H.

Therefore, our novel and simple ECG criterion, the V2S angle, seems to have a good AUC in the overall analysis. Since the number of LVOT cases was relatively small, the NPVs of most of the methods were low. It should be noted that the specificity and sensitivity parameters will not practically help physicians find the arrhythmia site. Predictive value is of special importance in clinics (19). The V2S angle showed a non-significantly higher positive predictive value for RVOT-origin arrhythmias than V2S/V3R or the TZ index (99.1% vs. 96.6% vs. 96.6%). Overall, V2S angle, V2S/V3R, and the TZ index have similar accuracies and can be used interchangeably.

The V2S angle is a quantitative measure of the proximity of the focus of the impulse to lead V2. In our study, the V2S angle was significantly higher in VAs originating from the RVOT. With a cut-off value of 58.28 mV°, the V2S angle predicted an RVOT origin with 85.7% sensitivity and 95.2% specificity (PPV: 99.1%; NPV: 52.6%). We thought that some factors, such as the patient’s physique, cardiac rotation, and respiratory variation, could be corrected by multiplying them by the anatomical cardiac long-axis. It is difficult to distinguish RVOT-originated VAs from LVOT-originated VAs with 100% precision. The QRS morphology of OTVAs can be affected by several factors, such as lead position, aortic deformities, obesity, the effect of medications, the effect on breasts in women, ventricular hypertrophy, chest wall deformities, and preferential conduction (16, 29, 30).

### Comparison with several angle-corrected ECG algorithms

Several ECG algorithms of previous studies were corrected by multiplying them by the angle in the retrospective cohort (*n* = 147). Surprisingly, the AUCs of the angle-corrected ECG algorithms for differentiating LVOT from RVOT were all higher than those before correction: The AUC of the V2S/V3R angle was higher than that of V2S/V3R (0.916 vs. 0.887). The AUC of the TZ index angle was higher than that of the TZ index (0.865 vs. 0.858). The AUC of the V1-2 SRd angle was higher than that of the V1-2 SRd (0.912 vs. 0.876). The AUC of the V1R/V1S index angle was higher than that of the V1R/V1S index (0.268 vs. 0.216). However, the AUC of the combination of the TZ index and V2S/V3R angle was lower than that of the combination of the TZ index and V2S/V3R (0.070 vs. 0.088) (Table 4). The AUC of the V2S/V3R index angle (AUC = 0.916) was the best index of all, even greater than that of the V2S angle (AUC = 0.888) (Figure 5). However, the V2S angle is simpler and more convenient than the V2S/V3R index angle.

Therefore, most of the previous ECG algorithms, corrected by the anatomical cardiac long-axis angle, can improve accuracy for differentiating LVOT- from RVOT-origin VA. This is an interesting and very meaningful discovery. Other angle-corrected ECG algorithms need to be further validated, such as the V3R/V7 index (31), the V4/V8 ratio (32), and the V1-V3 transition index (33).

### Clinical implications

Accurately predicting the origin of OTVT (the RVOT or LVOT) can optimize catheter ablation strategies, reduce ablation time, and reduce surgical complications. Therefore, the ability to more accurately predict an RVOT or LVOT origin of OTVA can have a significant impact on the ablation procedure.

In our study, under the condition of the same operator team and mapping system, the procedure time in the prospective cohort was shorter than that in the retrospective cohort due to the use of the V2S angle before RFCA (78.8 ± 31.8 min vs. 99.5 ± 40.8 min, *P* = 0.002). In particular, for predicting RVOT origin, the procedure time in the prospective cohort in which the V2S angle was used was significantly shorter than that in the retrospective cohort in which the V2S/V3R or the TZ index was used before RFCA (*P* = 0.004). However, there was no significant difference in the procedure time between the retrospective and prospective cohorts for predicting LVOT origin (*P* = 0.675) (Figure 7). This may be the reason for the small number of cases of LVOT origin. Therefore, future studies with larger sample sizes are needed.

It is well known that shortening the procedure time may improve RFCA efficiency and patient experience. However, it is worth noting that the procedure time may be affected by many factors, such as the operator’s surgical style, mapping system and coordination of the surgical team, and even the degree of patient cooperation. Therefore, the V2S angle can provide a rapid and accurate diagnosis of the OTVA origin before catheter ablation, allowing us to develop a better procedural strategy (decreasing ablation duration and radiation exposure) and avoid any unnecessary arterial or venous punctures. Of course, further study of other novel methods for accurately differentiating RVOT from LVOT as the site of origin of VA is required for reducing the rates of recurrence and unnecessary ablation applications. VCG when used as an alternative form of ECG provides important spatial information about the electrical activity of the heart, which achieves higher sensitivity in the detection of some pathologies, such as myocardial infarction, ischemia, and hypertrophy. It may be interesting to combine the analysis of the V2S angle with other methodologies based on the VCG method, the inverse ECG method, or machine learning methods, and this approach holds promise in providing electro-anatomic identification of the site of origin of focal premature depolarization.

### Study limitations

There were some limitations to this study. As a double-center study, the sample size was relatively small, and our results need to be confirmed in large, multi-center, prospective trials. Moreover, the algorithm used to identify the origin of the interventricular septum and the coronary sinuses cannot cover the whole RVOT (including the free wall) or LVOT (including the subaortic valve). Further studies with a larger patient population are needed to verify its range of application. Because of the retrospective nature of our study, we did not have the chance to investigate the confounding factors that affect the QRS morphology of OTVAs, such as lead position, aortic deformities, obesity, the effect of breasts in women, ventricular hypertrophy, chest wall deformities, and preferential conduction. Finally, outside of chest X-ray, we may be able to correct the position of the heart *via* cardiac color Doppler ultrasound, cardiac magnetic resonance, and other examinations and take advantage of machine learning technology (34) to attain clinical-grade precision in the prediction of the LVOT or RVOT as the origin of VT with fewer applicability restrictions.

## Conclusion

The V2S angle is a novel and simple ECG criterion for accurately differentiating RVOT- from LVOT-origin VA. The use of this simple ECG measurement could raise the accuracy of OTVA localization, and it could help decrease ablation duration and radiation exposure. This new method will hopefully improve mapping and ablation procedures.

## Data availability statement

The raw data supporting the conclusions of this article will be made available by the authors, without undue reservation.

## Ethics statement

The studies involving human participants were reviewed and approved by the Human Research Ethics Committee of Nanfang Hospital of Southern Medical University and the First Affiliated Hospital of Sun Yat-sen University. The patients/participants provided their written informed consent to participate in this study.

## Author contributions

SQ, ZS, XL, JL, XH, and YW contributed to the conception and design of the study. SQ, ZS, ML, LW, and YW organized the database. SQ, ZS, XL, JL, XH, JB, YL, and YX searched for and collected the relevant literature. SQ, ZS, XL, JL, XH, JX, and DZ performed the statistical analysis. SQ and ZS wrote the first draft of the manuscript. XL, JB, YL, JX, DZ, YX, LW, and YW revised and finalized the manuscript. All authors contributed to the manuscript revision, read, and approved the submitted version.
